# Risk Factors for Delayed Isolation of Patients with Active Pulmonary Tuberculosis in an Acute-care Hospital

**DOI:** 10.1038/s41598-019-41086-4

**Published:** 2019-03-19

**Authors:** Jaijun Han, Bo Da Nam, Se Yoon Park, Jebyung Park, Eunyoung Lee, Eun Jung Lee, Jung Hwa Hwang, Tae Hyong Kim

**Affiliations:** 1 Division of Infectious Diseases, Department of Internal Medicine, Soonchunhyang University Seoul Hospital, Seoul, 04401 Republic of Korea; 2 Department of Radiology, Soonchunhyang University Seoul Hospital, Seoul, 04401 Republic of Korea

## Abstract

The objective of the current study was to determine the factors associated with delayed isolation of pulmonary tuberculosis (TB). In this retrospective study, data of patients newly diagnosed with pulmonary TB from January 2015 through December 2017 at a referral hospital were reviewed. Delayed recognition of pulmonary TB was defined as failure to initiate airborne isolation within the first 3 days of admission. We analyzed the clinical, microbiological, and radiological factors associated with delayed isolation of pulmonary TB. A total of 134 patients with positive sputum acid-fast bacilli (AFB) cultures were analyzed, of which 44 (33%) were isolated within 3 days after admission. In multivariate logistic regression analysis, older age (p = 0.01), admission to departments other than Infectious Disease or Pulmonology (p = 0.005), and presence of malignancy (p = 0.02) were associated with delayed isolation. Patients with a radiologic diagnosis of active pulmonary TB were likely to be isolated early (p = 0.01). Better awareness of pulmonary TB among attending practitioners in hospital settings is required. Delay in isolation is associated with older age, malignancy, hospitalization to departments other than Infectious Disease or Pulmonology, and non-confident radiologic diagnosis of active pulmonary TB.

## Introduction

Tuberculosis (TB) is a major global health problem. Approximately 6.3 million new cases of TB and 1.3 million related deaths are recorded annually^1^. TB outbreaks are known to occur in institutional settings including hospitals^2^. In the Korean health care system, patient care during hospitalization is often communal, which can lead to an easy spread of TB^3^. In addition, immunocompromised patients who acquire TB as a nosocomial infection can develop severe disease. The World Health Organization’s policy on TB-infection control recommends administrative controls that reduce exposure to patients with presumptive TB^4^. Prompt diagnosis of active pulmonary TB is critical for early treatment and preventing disease transmission among patients and healthcare workers^5^.

In a previous study, the initial diagnosis of active pulmonary TB in almost 50% of the hospitalized patients was missed, and treatment was delayed for ≥7 days in 30% of the patients^6^. According to a systematic review, the diagnosis of pulmonary TB was delayed by an average of 68 days in low-income countries and 61 days in high-income countries^7^. A delay in TB treatment can result in progression and increased transmission of the disease^6^. Previous studies have reported certain factors associated with the delayed diagnosis of active pulmonary TB^8–10^. However, those studies were limited to a specific population^8^ or did not focus on hospitalized patients with a potential risk of causing in-hospital TB transmission^9,10^. Thus, knowledge of the factors associated with delayed isolation of patients with unexpected active pulmonary TB in acute-care hospitals is lacking.

This study aimed to determine the clinical and microbiological factors responsible for prolonged exposure before isolation of patients with TB in a health care setting. In addition, we evaluated the role of radiologists in the rapid diagnosis and isolation of patients with unexpected active pulmonary TB.

## Results

### Baseline demographics and clinical characteristics

**Figure 1:**
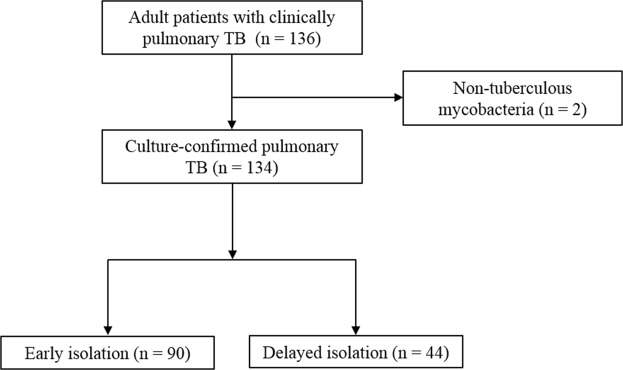
Flow diagram of the study.

During the study period (January 2015 to December 2017), a total of 134 patients with sputum cultures positive for *Mycobacterium tuberculosis* were identified, of which 44 (33%) had delayed isolation (Fig. 1). Table 1 shows the comparison of the baseline patient characteristics between the early and delayed isolation groups. Patients with delayed isolation were older than those with early isolation (mean 72 vs. 55 years, P < 0.001). A smaller proportion of patients with early isolation showed disease comorbidity (38% vs. 14%, P = 0.004), which included hypertension (32% vs. 52%, P = 0.03), chronic obstructive pulmonary disease (2% vs. 14%, P = 0.02), and malignancies (7% vs. 23%, P < 0.01) compared to patients with delayed isolation. In addition, patients with early isolation had more clinical symptoms and features of pulmonary TB such as cough, sputum, night sweats, and weight loss. These patients were more often admitted to the Infectious Disease or Pulmonology Departments than to other departments (76% vs. 41%, P < 0.001). The median number of hospitalization days were higher in patients with delayed isolation than in those with early isolation (10 vs. 19 days, P < 0.001). The overall in-hospital mortality was 13% (17/134), and in-hospital mortality was not significantly different between the two groups (12% in the early isolation group vs. 14% in the delayed isolation group, P = 0.82).

**Table 1:** Demographic and descriptive profiles of patients with pulmonary TB grouped according to the isolation period.

Characteristics	Total number of patients (n = 134)	Early isolation (within 3 days, n = 90)	Delayed isolation (after 3 days, n = 44)	p
Age, mean (SD)	61 (22)	55 (22)	72 (17)	<0.001
Male sex	90 (67)	65 (72)	25 (57)	0.08
**Comorbidity**				
None	40 (30)	34 (38)	6 (14)	0.004
Hypertension	52 (39)	29 (32)	23 (52)	0.03
Diabetes	41 (31)	25 (28)	16 (36)	0.31
COPD	8 (6)	2 (2)	6 (14)	0.02
Asthma	5 (4)	2 (2)	3 (7)	0.33
Cerebrovascular accident	11 (8)	7 (8)	4 (9)	0.75
Coronary artery disease	10 (7)	7 (8)	3 (7)	1.00
HIV	2 (2)	2 (2)	0 (0)	1.00
Chronic kidney disease	10 (7)	5 (6)	5 (11)	0.30
Malignancy	16 (12)	6 (7)	10 (23)	<0.01
TB history	18 (13)	12 (13)	6 (14)	0.96
TB medication history	14 (10)	9 (10)	5 (11)	0.77
**Clinical features**				
Fever	36 (27)	20 (22)	16 (36)	0.08
Chill	21 (16)	15 (17)	6 (14)	0.65
Cough	69 (52)	54 (60)	15 (34)	0.01
Sputum	43 (32)	35 (39)	8 (18)	0.02
Dyspnea	31 (23)	17 (19)	14 (32)	0.10
Chest pain	12 (9)	8 (9)	4 (9)	1.00
Other pain	21 (16)	9 (10)	12 (27)	0.01
Hemoptysis	6 (5)	5 (6)	1 (2)	0.66
Night sweats	10 (7)	10 (11)	0 (0)	0.03
Loss of appetite	24 (18)	19 (21)	5 (11)	0.16
Weight loss	23 (17)	21 (23)	2 (5)	<0.01
**Laboratory findings**				
Positive AFB smear	84 (63)	66 (73)	18 (41)	<0.001
Positive TB PCR	107 (87)	78 (93)	29 (74)	0.007
**Radiological findings (chest radiograph)**				
Diagnosis of TB	94 (70)	74 (82)	20 (46)	<0.001
Active TB	76 (57)	66 (73)	10 (23)	<0.001
Typical findings of TB in adults	76 (57)	61 (68)	15 (34)	<0.001
New disease on follow-up examination	37 (28)	25 (28)	12 (27)	0.04
**Hospitalization course**				
Admission to the Infectious Disease or Pulmonology Department	86 (64)	68 (76)	18 (41)	<0.001
Length of hospitalization (days, median (range))	13 (6–22)	10 (3–17)	19 (12–51)	<0.001
Exposure days, median (range)	1 (0–6)	0 (0–1)	9 (6–14)	<0.001
In-hospital mortality	17 (13)	11 (12)	6 (14)	0.82

Data are numbers (%) of patients, unless otherwise indicated.

Abbreviations: SD, standard deviation; COPD, chronic obstructive pulmonary disease; HIV, human immunodeficiency virus; TB, tuberculosis; AFB, acid-fast bacilli; PCR, polymerase chain reaction.

### Radiological findings

The radiological findings of pulmonary TB in our patients are presented in Table 2. Pulmonary TB was diagnosed by experienced radiologists after a retrospective review of chest radiographs in 70% (94/134) of the patients. In the remaining 40 patients, chest radiographic diagnoses were pneumonia (n = 17), bronchiectasis (n = 3), lung cancer (n = 2), pleural effusion (n = 2), nontuberculous mycobacterial disease (n = 1), pulmonary fibrosis (n = 1), septic lung (n = 1), lung abscess (n = 1), and nonspecific features including normal findings (n = 12). In the delayed isolation group, TB was retrospectively diagnosed on the basis of chest radiography in approximately half (45%, 20/44) of the patients. Furthermore, chest radiographic findings of active TB and typical findings of TB in adults were observed in only 23% and 34% of patients, respectively. On review of chest radiographs and CT scans, four patients (3%, 4/134) who showed negative findings on chest radiographs were subsequently diagnosed with TB by CT.

**Table 2:** Radiological findings of pulmonary TB.

Characteristic	Total number of patients (n = 134)	Early isolation (within 3 days, n = 90)	Delayed isolation (after 3 days, n = 44)	p
**Chest radiograph (n = 134)**				
Diagnosis of TB	94 (70)	74 (82)	20 (45)	<0.001
Active TB	76 (57)	66 (73)	10 (23)	<0.001
Typical findings of TB in adults	76 (57)	61 (68)	15 (34)	<0.001
New disease on follow-up examination	37 (28)	25 (28)	12 (27)	0.03
**Chest CT (n = 122)**				
Active TB	98 (73)	76 (84)	22 (50)	0.89
Typical findings of TB in adults	66 (49)	55 (61)	11 (25)	0.001
Airway involvement	22 (16)	16 (18)	6 (14)	0.59

Abbreviations: TB, tuberculosis; CT, computed tomography

### Factors associated with delayed isolation of TB

Table 3 shows the results of multivariate logistic regression for factors associated with delayed isolation with pulmonary TB during hospitalization. Patients of older age, with comorbid malignancy, and who were hospitalized in departments other than Infectious Disease or Pulmonology were significantly associated with delayed isolation. Patients who were diagnosed with active pulmonary TB on chest radiographs were more likely to be isolated earlier. The adjusted odd ratios after adjusting for potential confounders by excluding factors in the multivariate model subgroups of comorbidity, hospitalization course, and microbiological results are presented in Table 3.

**Table 3:** Multivariate logistic regression for factors associated with delayed isolation of patients with pulmonary TB during hospitalization.

Variables	Total		Model 1		Model 2		Model 3	
	OR (95% CI)	p*	OR (95% CI)	p	OR (95% CI)	p	OR (95% CI)	p
**Demographic**								
Age	1.04 (1.01–1.07)	0.01	1.04 (1.01–1.08)	0.002	1.04 (1.01–1.06)	0.01	1.04 (1.01–1.08)	0.01
**Comorbidity**								
Malignancy	4.75 (1.30–17.39)	0.02			5.95 (1.54–23.03)	0.01	4.86 (1.37–17.19)	0.01
**Clinical features**								
Other pain			4.11 (1.27–13.25)	0.02	3.97 (1.26–12.51)	0.02		
**Microbiological findings**								
AFB smear					0.31 (0.12–0.83)	0.02		
**Hospitalization course**								
Admission department	4.03 (1.52–10.74)	0.005	3.51 (1.39–8.85)	0.008			7.09 (2.34–21.46)	0.001
**Radiological findings (chest radiograph)**								
Activity	0.22 (0.08–0.66)	0.01	0.23 (0.08–0.64)	0.005	0.19 (0.07–0.57)	0.003	0.18 (0.06–0.52)	0.002

^*^All variables from Table 1 with P < 0.05 were included in the first step of multivariate analyses. Only significant variables in the final multivariate logistic regression model after backward selection are presented.

Model 1: Without comorbidity.

Model 2: Without hospitalization course.

Model 3: Without microbiological results.

Abbreviations: CI, confidence interval; SD, standard deviation; COPD, chronic obstructive pulmonary disease; HIV, human immunodeficiency virus; TB, tuberculosis; AFB, acid-fast bacilli; PCR, polymerase chain reaction.

## Discussion

Our study showed that older age, presence of comorbid malignancy, no confirmed diagnosis of active TB on chest radiograph, and admission to departments other than Infectious Disease or Pulmonology can contribute to the delayed isolation of patients with active pulmonary TB in an acute-care hospital. In addition, delayed isolation was associated with prolonged hospitalization and exposure days, which could lead to a potentially threatening hospital TB outbreak. On retrospective review of chest radiographs, pulmonary TB was diagnosed by experienced radiologists in 70% of all patients and approximately half (43%) of the patients in the delayed isolation group.

We found that older age was an important factor associated with delayed isolation, which was consistent with the results from previous population-based studies in developed countries^11,12^. In elderly patients, active pulmonary TB can manifest with unusual clinical features and co-exist with aspiration pneumonia^13^. According to 2017 nationwide data from the Korea Centers for Disease Control and Prevention, 41.9% of newly diagnosed TB cases were patients aged ≥65 years, and this value has been increasing. The number of patients with newly diagnosed TB who are aged ≥80 years has been increasing since 2001^14^. In an aging society, elderly patients with unusual symptoms or signs should be examined for possible TB infection. In the population aged ≥65 years, 1 in 10 persons has a malignant disease^15^. In this study, malignancy was a risk factor for delayed isolation. In the delayed isolation group, approximately one-fourth (22.72%, 10/44) of our patients had comorbid malignancies. Therefore, TB should be considered in the differential diagnosis of hospitalized patients, particularly in elderly patients with malignancy.

In this study, the initial department of admission for each patient was associated with a delayed diagnosis of TB. In previous studies, the type of hospital on initial visit or the level of the attending practitioner was associated with a delayed diagnosis of TB. Supplemental Table 1 show the detailed cause of admission or presenting symptom/sign. Most common department other than Infectious Diseases or Pulmonology was Gastroenterology followed by Orthopedic, Nephrology, and Thoracic surgery (see Supplemental Table 2). Among the patients who admitted to departments other than Infectious Diseases or Pulmonary, 54% (26/48) patients had delayed isolation. Of these 26 patients, 5 had extrapulmonary TB such as TB spondylitis (n = 3), meningitis (n = 1), and peritonitis (n = 1). In patients with extrapulmonary TB, physician should carefully check whether they are accompanied by pulmonary TB and isolate if necessary.

Patients with delayed isolation had symptoms indicating diseases other than pulmonary TB, such as pneumonia, lung mass, urinary tract infection, and liver cirrhosis with complications (see Supplemental Table 3). These patients had clinical features of systemic infectious disease, but the diagnosis of TB was delayed, possibly because of low AFB smear yield and decreased burden of pulmonary TB. Among the 44 patients who had delayed isolation, active pulmonary TB was diagnosed from the specimen collected during bronchoscopy (n = 6), subsequent chest CT (n = 7), and sputum specimen collected for evaluation of other disease such as pneumonia (n = 8). This result suggests that initial admission to an inappropriate department can delay the detection of active pulmonary TB and contribute to prolonged in-hospital airborne exposures.

Previous reports describe factors associated with a diagnostic delay of pulmonary TB^8,9,16–18^. In a Japanese study, male gender, absence of chronic cough, and presence of non-cavitary lesion on chest radiograph were associated with delayed isolation of patients with smear-positive pulmonary TB^8^. However, the Japanese study was limited by its inclusion of only AFB smear-positive patients. The present study included all patients with active TB who were isolated to prevent further transmission and reduce the burden of infection control. Pulmonary TB was confirmed by culture positivity in all our patients, but 50 (37%) patients had negative AFB smear results. Thus, negative AFB smear results might not reveal a patient who has contagious active pulmonary TB that can be transmitted to others and lead to a possible outbreak^19^.

This study highlights the importance of chest radiographic information and diagnosis by a radiologist for patients with pulmonary TB. A chest radiograph with pulmonary TB may be inaccurately interpreted by a physician^20^. Recent studies have reported that high-resolution CT screening is useful for detecting culture positive pulmonary TB^21,22^. However, those image studies have limited diagnostic performance when clinical information is lacking. In the present study, we found that 46% of our delayed isolation group were retrospectively diagnosed with pulmonary TB by chest radiologists during review of the examination results. In addition, confirmed active pulmonary TB by a radiologist was significantly associated with early isolation. Therefore, we expect that rapid interpretation of chest radiograph by a radiologist can reduce an unexpected exposure period. Because this interpretation is inferred by retrospective review of chest radiographs, the role of the radiologist in reducing an unexpected exposure to pulmonary TB needs to be evaluated in prospective studies.

Despite our important findings, there are a few limitations to this study. First, our study was based on a single-center cohort. Therefore, the clinically delayed decisions leading to exposure before isolation might have been made by clinicians at differing levels of experience, and our results may not be generalized to other institutions with different prevalence or incidence rates of TB. Second, owing to the retrospective nature of the study, the accuracy of documented charts is limited. Further prospective studies that involve multiple institutions are required to confirm our findings.

Although the incidence of pulmonary TB is decreasing worldwide, pulmonary TB outbreaks are reported consistently. These cause disease transmission and increasing hospital costs. Therefore, practitioners should be more careful and aware of the factors associated with delayed isolation of patients of older age, with malignancy, hospitalized to departments other than Infectious Disease or Pulmonology, and showing uncertain activity of pulmonary TB on chest radiography.

## Methods

### Study setting and population

This retrospective study included all hospitalized patients who were diagnosed with culture-positive active pulmonary TB at a university-affiliated referral center in Seoul, Republic of Korea, between January 2015 and December 2017. At our institution, patients with respiratory diseases such as pulmonary TB are usually admitted to the Pulmonology or Infectious Disease Department. If a patient is initially suspected of active pulmonary TB, preemptive airborne isolation with negative air pressure is performed at admission. If it is unclear whether the patient has active pulmonary TB, the patient can be isolated under suspicion, depending on the physician’s clinical decision. This study was approved by the Institutional Review Board (serial number 2018-04-033) of the hospital, and the need for informed consent was waived owing to the retrospective nature of the study.

### Data collection and definitions

Electronic medical records of patients diagnosed with active pulmonary TB were reviewed. A diagnosis of active pulmonary TB was confirmed via AFB culture of respiratory specimens positive for *Mycobacterium tuberculosis*. We divided the patients with active pulmonary TB into the early or the delayed isolation group based on being isolated within 3 days, or after 3 days, of hospitalization respectively^23,24^. We focused on factors that might contribute to delayed isolation of such patients after 3 days of admission, which could lead to continued exposure during their hospital stay. We collected data on potential confounders such as patient-related demographics (age, sex, chief complaints, presenting symptoms and signs of illness, past medical history, and admission department), microbiological results (AFB staining and culture, and TB DNA polymerase chain reaction test), and radiologic findings (chest radiograph and/or chest computed tomography). Axillary measured body temperature >37.8 °C was considered a fever. Weight loss of ≥10% of body weight or ≥5 kg in the last 6 months was noted. Malignancies included all solid and hematologic types. The time from admission to adequate airborne isolation was defined as the exposure period.

### Evaluation of radiological findings

The initial chest radiographs (134/134) and CT scans (122/134) of enrolled patients were reviewed by two chest radiologists, one with 26, and the other with 7, years of experience. Radiological diagnosis, including that of TB, and evaluation for disease activity, were performed without clinical information to eliminate preconceived bias. Final decisions were reached by consensus. Radiological findings of active pulmonary TB included cavities, nodules, and micronodules with segmental distribution, airspace consolidation, diffuse miliary nodules, and new appearance or progression of these findings on follow-up when the previous study was available. Findings of stable or inactive pulmonary TB included absence of active disease, mostly calcified small granulomas or lymph nodes, and stable fibronodular scarring for at least 6 months. Radiological diagnosis of TB activity was classified as active, inactive, and indeterminate. In addition, typical and relatively atypical radiological manifestations of TB in adults were evaluated. The typical findings included upper lung involvement, cavities with nodules or micronodules, and unilateral pleural effusion suggesting TB pleurisy. The atypical findings included middle and lower lung involvement, lobar or segmental airspace consolidation, diffuse miliary disease, bilateral pleural effusions, lymphadenopathy with no definite lung diseases, and airway involvement^25,26^.

### Statistical analysis

Continuous variables were compared using the non-parametric Mann-Whitney and Kruskal-Wallis tests. Categorical variables were compared using Chi-square test or Fisher’s exact test. Factors possibly associated with early TB diagnosis were identified using univariate analysis (p < 0.05) and entered into multivariate models. The final model was selected using backward selection, and factors that did not improve the adequacy of the model were discarded. In addition, we have implemented virtual settings for clinically important variables with three situations by adjusting all significant values without comorbidity (model 1), hospitalization course (model 2), and microbiologic results (model 3). Data were analyzed using SPSS version 19.0 (Statistical Package for the Social Sciences Inc.; Chicago, IL, USA). Variables with a P-value of <0.05 were considered to be statistically significant.

## Supplementary information

Dataset 1

## Data Availability

The datasets generated during and/or analyzed during the current study are available from the corresponding author on reasonable request.
