# Supplementary material for: Risk Factors for Delayed Isolation of Patients with Active Pulmonary Tuberculosis in an Acute-care Hospital
Source: Sci Rep. 2019 Mar 19;9:4849. doi: 10.1038/s41598-019-41086-4 (PMC6424955; doi:10.1038/s41598-019-41086-4)
Supplement: Supplementary file 1 — Dataset 1 [file 41598_2019_41086_MOESM1_ESM.docx]

# Supplementary Information

***Risk Factors for Delayed Isolation of Patients with Active Pulmonary Tuberculosis in an Acute-care Hospital***

Jaijun Han^1,+^, Boda Nam^2,+^, Se Yoon Park^1,*^, Jebyung Park^1^, Eunyoung Lee^1^, Eun Jung Lee^1^, Jung Hwa Hwang^2^ and Tae Hyong Kim^1^

(^*^corresponding author)

^1^Division of Infectious Diseases, Department of Internal Medicine, Soonchunhyang University Seoul Hospital, Seoul, 04401, Republic of Korea.

^2^Department of Radiology, Soonchunhyang University Seoul Hospital, Seoul, 04401, Republic of Korea

**Supplemental Table 1.** Cause of admission for 48 patients who were admitted to departments other than infectious diseases or pulmonology.

| Cause for admission or presenting symptom/sign | Total, n=48 | Delayed isolation, n=26 |
| --- | --- | --- |
| TB managed by international clinic | 5 (10) | 1 (4) |
| Infectious spondylitis (TB spondylitis) | 4 (8) | 3 (12) |
| Fracture (Femur, Patella, and Distal radius) | 3 (6) | 1 (4) |
| Urinary tract infection | 3 (6) | 3 (12) |
| Pneumonia | 3 (6) | 2 (8) |
| For operation work up (Spinal stenosis, Rectal cancer, Vascular access) | 3 (6) | 2 (8) |
| Trauma | 2 (4) | 0 |
| Pancreatitis | 2 (4) | 1 (4) |
| Abdominal pain | 2 (4) | 1 (4) |
| Pneumothorax | 2 (4) | 2 (8) |
| Cholecystitis/cholangitis | 2 (4) | 1 (4) |
| General weakness, weight loss | 2 (4) | 0 |
| Ascites control | 1 (2) | 1 (4) |
| Hyponatremia | 1 (2) | 1 (4) |
| TB meningitis, hyponatremia | 1 (2) | 1 (4) |
| Peritonitis (TB peritonitis) | 1 (2) | 1 (4) |
| Aortic (Intramural) hematoma | 1 (2) | 1 (4) |
| Reflux esophagitis | 1 (2) | 1 (4) |
| Hepatocellular carcinoma | 1 (2) | 1 (4) |
| Ischemic colitis | 1 (2) | 0 |
| Cellulitis | 1 (2) | 0 |
| Gastritis | 1 (2) | 0 |
| Brain hemorrhage | 1 (2) | 0 |
| Rectal cancer re-staging | 1 (2) | 0 |
| RA flare | 1 (2) | 1 (4) |
| Tremor | 1 (2) | 0 |
| On TB medication (transfer) | 1 (2) | 0 |

Data are numbers (%) of patients.

Abbreviations: TB, tuberculosis; RA, rheumatoid arthritis.

**Supplemental Table 2.** Departments for 48 patients who were admitted to departments other than Infectious Diseases or Pulmonology.

| Admission department | Total, n=48 |
| --- | --- |
| Gastroenterology | 13 (27) |
| Orthopedics | 8 (17) |
| Nephrology | 5 (10) |
| Thoracic surgery | 5 (10) |
| Neurosurgery | 4 (8) |
| Family medicine | 4 (8) |
| Neurology | 3 (6) |
| Hematology | 2 (4) |
| General surgery | 2 (4) |
| Cardiology | 1 (2) |
| Rheumatology | 1 (2) |

Data are numbers (%) of patients

**Supplemental Table 3.** Causes of hospitalization in patients with delayed isolation (n=44)

| Disease criteria or cause of admission | Total, n=44 |
| --- | --- |
| Pneumonia | 15 (34) |
| Lung mass evaluation | 3 (7) |
| Urinary tract infection | 3 (7) |
| Complications of liver cirrhosis | 3 (7) |
| Gastroenteritis | 3 (7) |
| Osteomyelitis/discitis | 3 (7) |
| Pneumothorax | 2 (5) |
| Aggravation of IPF/COPD | 2 (5) |
| Arthritis/knee injury | 2 (5) |
| Cellulitis | 1 (2) |
| Cholecystitis | 1 (2) |
| Pancreatitis | 1 (2) |
| Cancer radiotherapy | 1 (2) |
| Rectal cancer operation | 1 (2) |
| Vascular access operation | 1 (2) |
| Aortic (intramural) hematoma | 1 (2) |
| Metabolic encephalopathy | 1 (2) |

Data are numbers (%) of patients

Abbreviations: TB, tuberculosis; IPF, idiopathic pulmonary fibrosis; COPD, chronic obstructive pulmonary disease
